# Exploring the Correlation Between the Regulation of Macrophages by Regulatory T Cells and Peripheral Neuropathic Pain

**DOI:** 10.3389/fnins.2022.813751

**Published:** 2022-02-14

**Authors:** Hongyu Chen, Liangfu Jiang, Dupiao Zhang, Jianpeng Chen, Xiaobin Luo, Yutong Xie, Tao Han, Liang Wang, Zhe Zhang, Xijie Zhou, Hede Yan

**Affiliations:** ^1^Division of Hand Surgery, Department of Orthopedics, The Second Affiliated Hospital and Yuying Children’s Hospital of Wenzhou Medical University, Wenzhou, China; ^2^Key Laboratory of Orthopedics of Zhejiang Province, Wenzhou, China; ^3^The Second School of Medicine, Wenzhou Medical University, Wenzhou, China

**Keywords:** regulatory T cells, macrophage type M1/M2, neuropathic pain, traumatic neuroma, peripheral nerve

## Abstract

**Objective:**

Intractable pain after peripheral nerve injury has become a major concern in the field of pain. Current evidence shows that routine medications or surgical treatment is associated with inconsistent results and different curative effects. Stable and effective treatment methods in clinical practice are also lacking. To date, there is no consensus on the pathophysiological mechanisms of pain. The present study investigates the potential regulatory role of regulatory T cells in the differentiation of macrophages on dorsal root ganglion (DRG) and explores the mechanism of nociceptive signals in the signal transfer station. The findings are expected to guide the prevention of various types of peripheral neuropathic pain.

**Methods:**

Thirty-six male Sprague Dawley (SD) rats and 18 male Nude rats, of equal weight (250–300g), were used in this study. The rats were divided into 3 groups: SD rat sciatic nerve transection group (SNT group, *n* = 18), SD rat nerve transection experimental group (SNT/RAPA group, *n* = 18) and Nude rat nerve transection experimental group (SNT/NUDE group, *n* = 18). The behavior related to neuropathic pain of animals were comprehensively evaluated in all groups. Furthermore, we analyzed the degree of neuroma development, histology, gene, and protein expression, and compared their correlation with the ultrastructural changes of M1/M2 type differentiation of macrophages in DRG.

**Results:**

Sciatic nerve transection (SNT), induced the aggregation of several types of macrophages in lumbar DRG of SD rats leading to a higher ratio of M1/M2. Following the inhibition of the M1 type polarization of macrophages, axon outgrowth increased significantly. A significantly lower average autotomy score was reported in the SNT/NUDE group (*p < 0.05) and the SNT/RAPA group (^@^*p* < 0.05) as compared to that of the SNT group. The SNT/NUDE group showed no noticeable neuroma formation 30 days after the nerve transection. However, bulbous neuromas were observed in the nerve stumps of both the SNT control and SNT/RAPA groups. Immunofluorescence staining revealed a significant decrease in the proportion of M1/M2 macrophages in lumbar DRG of the SNT/NUDE group (^**^p < 0.001) and the SNT/RAPA group (^@^*p* < 0.05) compared to the SNT group. The expression of pain-related proteins was also decreased (^@^*p* < 0.05, *p < 0.05,^**^p < 0.001). Also, the expression of alpha-smooth muscle actin (α-SMA), neurofilament 200 (NF-200), and nerve growth factor low-affinity receptor p75 were significantly down-regulated in the nerve tissue (^@^*p* < 0.05, ^@@^*p* < 0.001, ^**^p < 0.001).

**Conclusion:**

M1/M2 type differentiation of macrophages on DRG plays a significant role in the formation of traumatic painful neuroma after neurotomy. In combination with our previous study, the results of this study suggest that regulatory T cells reduce the ratio of M1/M2 macrophages and alleviate the pain of neuroma by regulating the polarization direction of macrophages on neuroma. These findings provide key insights into developing new strategies to manage painful neuroma.

## Introduction

Despite the advancement of modern industrial civilization, the recent past has seen an unprecedented rise in trauma and amputation caused by natural and unnatural factors (Bowsher, 2002; Bonfiglioli et al., 2015). While limbs are faced with various soft tissue injuries, the peripheral nerves also suffer different types and varying degrees of injury, among them, nerve crush injury and nerve severance injury (Noble et al., 1998). The American Pain Association, in the past month, projected that nearly half of Americans will experience pain lasting at least one day (Macfarlane, 2016). Peripheral neuropathic pain has the characteristics of refractory, repetitive, and intractable, which brings great physical and mental pain to patients (Zimmermann, 2001). Such pain response is often accompanied by symptoms of autonomic dysfunction associated with a severe impact on the psychological mood and quality of life of individuals. Pain also may increase the social burden and has become a major societal problem (Bowsher, 2002; Yan et al., 2012). Although more and more factors that contribute to the pain perception in response to peripheral nerve injury have been discovered, no consensus has been reached on the precise pathophysiological mechanism of pain (Mackinnon, 1997). Scholars hold different views on the mechanism of pain. Drug therapy or surgical treatment yield unsatisfactory results with varying curative effects, which is why there is a need for more stable and effective treatment methods (Zeilhofer et al., 2011; Yao et al., 2017).

**FIGURE 1:**
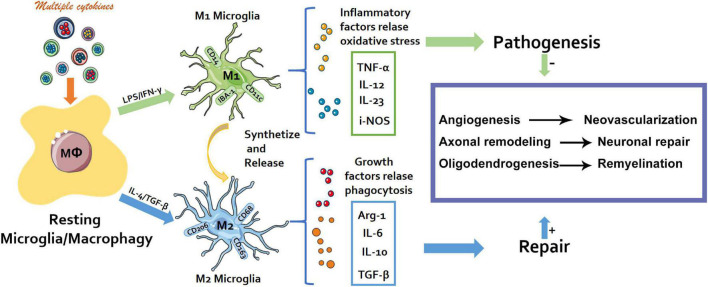
Schematic diagram. The M1/M2 polarization process of macrophages and their different physiological effects.

Rapamycin (RAPA) is a macrolide drug, which produces inhibitory effect through mammalian rapamycin (mTOR) target. It is widely used in the treatment of graft rejection and cancer. Studies have shown that Rapa inhibits the proliferation and function of effector T cells (Teff) *in vivo* and *in vitro* (Chen et al., 2021). Compelling evidence has demonstrated how immune response is pivotal in the development of neuropathic pain (Ji et al., 2016; Malcangio, 2019). In particular, the neuroinflammation of the peripheral nervous system and central nervous system induced by immune cell activity mediates the occurrence, development, and maintenance of chronic neuropathic pain (Walters, 2014; Ji et al., 2016). Macrophages (Mφ) are a crucial component of innate immunity (Crotti and Ransohoff, 2016). In the recent past, the major focus of research has been on the origin and function of macrophages, and their role in disease promotion or protection (Ji et al., 2016). The components of the microenvironment, especially cytokines induces macrophage polarization into M1/M2 type macrophages, in the manner that they express corresponding specific genes and perform different functions (Figure 1) (Chen et al., 2018; Zigmond and Echevarria, 2019).

Neurons of the dorsal root ganglion (DRG), the main afferent neurons of the peripheral nervous system, are the transfer station via which nociceptive signals are transmitted to the central system (Esposito et al., 2019). The peripheral ending of a DRG neuron with the cell body situated in a DRG is activated and the action potential (nerve signal) passes from the peripheral to the central process via the T-junction (Aldskogius and Kozlova, 2013). External factors, including cutting, tearing, or squeezing may injure the peripheral nerves, breaking the nerve fibers and cause inevitable Wallerian degeneration of the nerve fiber ends (Simons et al., 2014). The occurrence of Wallerian degeneration triggers the transmission of neuro-electrophysiological signals to the DRG via the nerve fibers and enters the posterior horn of the spinal cord. Consequently, the corresponding nerve tissue experiences a decline or loss of related functions (Beirowski et al., 2005).

Our previous investigation revealed aggregation of several types of macrophages in the DRG of the spinal cord of ordinary SD rats at different stages of injury, which implied a higher ratio of M1/M2 (Ji et al., 2016). A comprehensive assessment of the SNT/NUDE group 30 days after the neurotomy demonstrated that the rats had significantly low pain and did not have a traumatic neuroma. M1 type polarization of macrophages in the rat DRG was suppressed following treatment of SD rats with an immunosuppressant (rapamycin), whereas the M2 type polarization was up-regulated significantly. Moreover, the SNT/RAPA group exhibited: (i) down-regulated expression of nerve growth factor receptor p75, α-SMA, and NF-200; (ii) a significant increase in the natural growth of axons; (iii) no obvious neuroma formation; (iv) decreased expression of pain protein. Although Monika Sharma et al. reported the molecular mechanism, but in nociceptive signal transduction of peripheral neuropathic pain is still elusive (Sharma et al., 2020). Findings from this work will provide a solid theoretical basis for future clinical practice and block drug research.

## Materials and Methods

### Animal Models and Grouping

This study was approved by the Ethics Committee of Wenzhou Medical University (Approval: wydw2019-0954). All rats were purchased from the Experimental Animal Center of Wenzhou Medical University. Rats were treated following the guidelines of the National Research Council on the care and use of laboratory animals and allowed free access to food and water. Thirty-six male SD rats and 18 male nude rats of the same weight (250-300g) were used to construct sciatic nerve dissection models. The rats were divided randomly into 3 groups: SD rat control group (SNT group, *n* = 18), SD rat nerve dissociation experimental group (SNT/RAPA group, *n* = 18), and nude rat nerve dissociation experimental group (Nude rat experimental group, *n* = 18).

### Surgical Procedure

**FIGURE 2:**
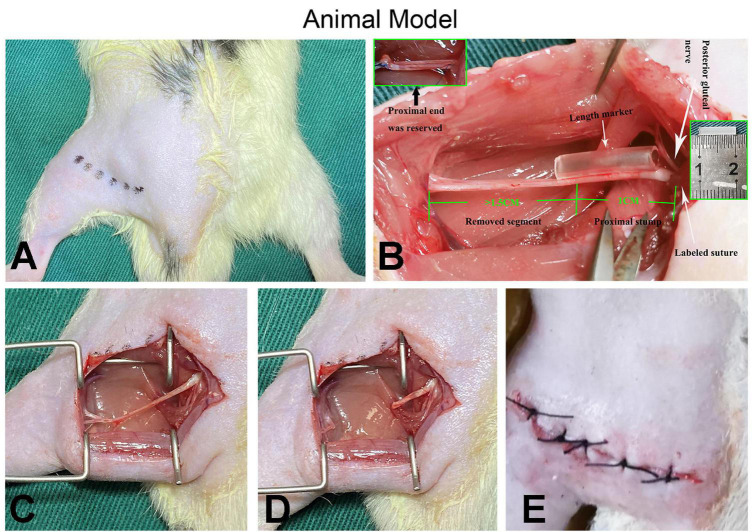
Demonstration of surgical procedures. **(A)** After the animal is shaved, the surgical entry position (the muscle gap between the biceps femoris and the gluteal muscles) is marked, the surgical area is disinfected, and draped. **(B)** The white arrow on the right indicates the origin of the posterior gluteal nerve (top) and the location where the marked sutures are placed for quantitative analysis (bottom); the white arrow in the middle indicates the length marker (silicone catheter in the middle) for precise nerve cutting. The green numbers 1 and 1.5 and the illustrations (top left and middle right) show the details of the preparation of the proximal nerve stump. **(C–E)** The muscle wound and the skin incision are sutured with 4-0 sutures after the preparation process and completion of the PNS (proximal nerve stump).

The surgery was conducted following a protocol described in our previous research (Yan et al., 2014). Each rat was anesthetized via intraperitoneal injection of sodium pentobarbital (50 mg/kg). The left sciatic nerve was exposed between the biceps femoris and gluteal muscles under sterile conditions. The position of the posterior gluteal nerve branching around the sciatic nerve notch level was located under a microscope and marked with 7-0 suture to perform the quantitative analysis of neuroma growth. Assisted by a marker (1 cm long), a sharp cut was made on the sciatic nerve 1 cm distal to the marked site and separated from the surrounding tissues up to the marked site. To avoid spontaneous nerve regeneration, a gap of at least 1.5 cm was maintained at the distal end of the traverse point in all cases. Muscle wounds and skin incisions were sutured with 4-0 sutures after hemostasis and placed in cages, respectively (Figure 2). All surgeries were performed by the same group of experienced surgeons under an operating microscope; ibuprofen was administered daily as postoperative analgesia for 1 week. According to published literature, the SNT/RAPA group was intraperitoneally injected with rapamycin [AY-22989, 2.0mg/(kg × day)] at 1, 4, 7, 10, 13, 16, 19, 22, 25, and 28 days post-surgery, whereas the SNT/NUDE group and the SNT group were injected with the same volume of normal saline for 28 days (Zhang et al., 2018; Calabrese et al., 2020; Zhou et al., 2020).

### Behavior and Gait Analysis

For each group of rats (*n* = 18) their autotomy scores were monitored within 30 days. Double-blind observers utilized the modified Wall scale to record the number of points based on the severity of autotomy. The autotomy score was recorded thrice a week to assess the degree of neuropathic pain as follows: (Zeltser et al., 2000) 1 point is assigned to 2 or more toenails (maximum, 1 point per limb), 1 point is assigned to each half finger (distal and proximal phalanx), and each limb can attain a maximum of 10 points. Animal gait analysis system (CatWalk XT), a complete gait analysis system whose core component is a walking platform, was used for quantitative evaluation of the steps and movements of rats. On the 30^th^ day post-surgery, each group of rats was allowed to walk across the walking platform and recorded in real-time. In the system, footprint bright refraction technology captures the real footprints utilizing a high-speed camera placed under the walking platform. The automatic classification technology of footprints was employed to enable the system to automatically classify the footprints into the front left, back left, front right, and back right. The intensity of the footprint, the time and distance between the footprints, and other relevant statistical information in the animal model was determined by measuring the size of the footprint. A similar approach can be used to evaluate motor coordination and automate, fast, objective and complete analysis of changes related to gait after neurotraumatic pain (Heinzel et al., 2020).

### Neuroma Growth Assessment

To quantify the growth differences between the groups, the nerve stump was marked 1 cm proximal to the traverse point with 7-0 sutures. The sciatic nerve was transected from the previously marked point on the surgical site after the experiment. Subsequently, the corresponding ganglion segment (1 cm long) was cut from the contralateral side to quantify neuroma growth. Using the weight ratio (WR), the growth of neuroma was evaluated using the formula: [NW (weight of neuroma)–NNW (weight of normal nerve segment removed)]/NNW × 100%.

### Histological Analysis

The proximal nerve stump was excised and all tissue specimens fixed in 4% paraformaldehyde overnight and processed via standard paraffin embedding methods. Longitudinal sections (5 μm thick) of the proximal nerve stump were assessed via H&E and Masson’s trichrome staining methods. The H&E staining was performed on sections of rat organs. An optical microscope (Nikon Eclipse 80i, Tokyo, Japan) was used to acquire micrographs of the stained samples. Three fields of view in each part were selected randomly, recorded, and analyzed. Immunohistochemistry was performed following standard procedures. Specimens were collected on the 30^th^ experimental day (Zhou et al., 2020). The nerve tissue was stained with anti-NF-200 (labeled axons), anti-p75 (NGF receptor), and anti-α-SMA. DRG in the left fourth segment of the lumbar spine (L4),which was double-stained with anti-IBA-1 (labeled M1 macrophages) and anti-CD206 (labeled M2 macrophages), respectively, anti-rabbit IgG conjugated with Alexa Fluor 488 and anti-mouse conjugated with Alexa Fluor 594 IgG incubation. The statistical analysis of immunofluorescence staining were randomly selected and measured from 3 fields in each section. There are a total of 9 sections with 3 animals in each group. A Nikon confocal laser microscope (Nikon, A1 plus, Tokyo, Japan) or Nikon Eclipse 80i fluorescence microscope was employed to capture images. To calculate for different antibody immunoreaction positive areas (%), the IOD (the integrated option density) formula, value/selected area*100%, was used. Image J software was applied to measure the IOD and the area of the selected part. All immunohistochemical supplies, including primary antibodies, were purchased from Sigma (Sigma, United States).

### Transmission Electron Microscopy Examination

Specimens were immediately fixed with 2.5% glutaraldehyde in 0.1M phosphate buffer overnight. Subsequently, the specimens were fixed with 1% samarium tetroxide, stained with uranyl acetate, acidified, and dehydrated with 2,2-dimethoxypropane, followed by epoxy resin embedding. Ultra-thin sections (70 nm, color silver flakes) were cut using an LKB8800 ultra-thin microtome, pasted on the grid, and examined under a transmission electron microscope. Image J software was employed for quantitative assessment of each segment of 10 random field nerve sections, including morphometric indicators (such as the thickness of myelin sheath), the ratio of myelinated nerve fibers to myelinated nerve fibers, and the percentage of fibroblasts to the total number of cells. All measurements were performed by investigators blinded to the grouping information for each part.

### Western Blot Analysis

Tissue specimens were lysed in a lysis buffer (100 mmol/L dithiothreitol, 50 mmol/L Tris-HCl, pH 6.8, 2% SDS, and 10% glycerol) containing protease inhibitors. Total protein concentration was assessed via the BCA method. The total protein was kept at −80°C awaiting subsequent analysis. Equal amounts of proteins were separated via sodium dodecyl sulfate-polyacrylamide gel electrophoresis (SDS-PAGE) technology and transferred to PVDF (polyvinylidene fluoride) membrane. 5% diluted skimmed milk powder was used as a sealant and imprinted in Tris-Buffer salt water for 3 h at a time. Rabbit anti-substance P (dilution: 1:500, GeneTex, Inc., United States) and mouse anti-c-fos (dilution: 1:200, Santa Cruz Biotechnology, United States) monoclonal antibodies were blotted at 4°C for overnight incubation. After washing, the membrane was incubated with conjugated goat anti-mouse IgG (1:8000) or goat anti-rabbit IgG (1:5000) for 2h at room temperature. Next, the membrane was washed with buffer. Immune response bands were examined using the ChemiDoc XRS + Bio-Rad imaging system. Image J software (National Institutes of Health) was applied to determine the quantitative density of the membrane, with beta-actin (dilution: 1:1000, Santa Cruz Biotechnology, United States) as the control.

### Thermal Pain Measurement

The hot and cold pan pain tester [BIO-CHP] (Bioserve, France), is used to assess the heat sensitivity of animals to pain induced by exposure to heat or cold. The main structure of the Bioseb Thermal Analgesia Tester includes a metal plate that can be heated to 65°C and cooled to −4°C (applicable ambient temperature is 20°C to 25°C). After testing, we selected an optimal temperature (55°C) that would not cause damage to the rat and just make it feel the thermal stimulus to lift the hind paw to evaluate the reaction time of the rat. Animals were placed on the surface of the metal plate, and the heating or cooling steps were initiated. The built-in timer was used to determine the animal’s sensitivity to heat and cold stimulation. Upon realizing that the animal felt the stimulus and lifted its hind paw, the timing was stopped, and the time taken for the animal to react was monitored and recorded. The response time of the animal was employed as a consideration of the pain resistance of the animal and applied in the analgesic effect test.

### Statistical Analysis

All statistical analyses were performed in SPSS software version 19.0 (SPSS, Chicago, Illinois). Data were expressed as mean ± standard error (SEM). Independent sample test and one-way analysis of variance [LSD (assumed equal variance) or Dunnett’s T3 (not assumed equal variance)], were applied for statistical analysis between groups. A P value less than 0.05 denoted statistical significance.

## Results

### Autotomy Score and Gait Observation

**FIGURE 3:**
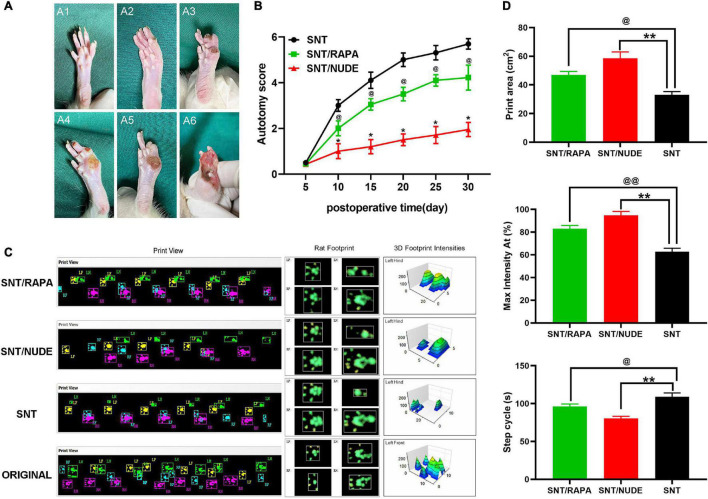
Scoring results of autotomy behavior and gait observation and analysis. **(A)** Modified Wall’s score for rats. No autotomy (score 0) (A1); loss of half of the second toe (score 1) (A2); loss of the first and the third toes (score 1) or complete loss of the second toe (score 2), score 1 + 2 = 3 totally (A3); loss of the first and second toes (score 2 + 2 = 4) (A4); loss of the first and the second toes, half of the third toe, the total score of 2 + 2 + 1 = 5 (A5); the first, second and third toes are missing, the total score is 2 × 3 = 6 (A6). **(B)** At all time points except the 5^th^ day (*p* > 0.05), the autotomy score between the SNT group and the other 2 experimental groups is statistically significantly different (*p < 0.05, ^@^*p* < 0.05). **(C)** CatWalk XT recorded real-time walking footprints of each group of experimental animals on the 30^th^ day after surgery (left), representative footprints of rats (middle), and detected the 3D signal intensity of footprints (right). **(D)** Quantification of Print area (cm^2^), Max intensity At (%) and Step cycle (s) in each group (^@^*p* < 0.05, ^@@^*p* < 0.01, **p < 0.01).

The surgical side (left foot) was assessed for all animals and their autotomy behavior was recorded within 5 to 30 days after the surgery at every other time point except for the 5^th^ day. The average autotomy score between the SNT/RAPA group and the SNT group was statistically significantly different (*p* < 0.05). The average autotomy score between the SNT/NUDE group and the SNT group was also statistically significantly different (*p* < 0.05). In addition, the average autotomy score of the SNT/NUDE group was significantly lower compared to that of the SNT group and SNT/RAPA group (Figures 3A,B). Of the 18 rats in the SNT group 15 (83.3%) exhibited different degrees of autotomy (score 3–6 points, average 5.69 ± 0.23 points), whereas, in the SNT/RAPA group, 11 rats (61.1%, score 2 ∼5 points, average 4.22 ± 0.54 points) exhibited autotomy behavior. Four of the 18 rats in the SNT/NUDE group (22.2%, autotomy score 1-3 points, average 1.95 ± 0.31 points) showed signs of autotomy. According to the animal gait analysis system, all animals in each group crossed the T stage without any problems and they used all 4 paws. The printed view of the walking footprints of each group of experimental animals was recorded on the 30th day after surgery in real-time using CatWalk XT. However, the gait speed and gait frequency of the three groups of experimental animals were not significantly different. Rat footprints revealed that rats in the SNT group exhibited a significantly larger gait area on the right front, right back, and left front, which is manifested as an increase in step length and step width as compared to normal rats without surgery. The gait imprints of these two experimental rats, particularly the SNT/NUDE group, were closer to those of normal rats. The 3D footprint signal strength monitoring data also provided strong evidence on the above experimental results (Figure 3C). The quantitative analysis results of Print area (cm^2^), Max intensity At (%) and Step cycle (s) of rats in each group are shown in that (Figure 3D).

### Gross Evaluation

The SNT/NUDE group showed a significantly lower weight ratio of neuroma (0.770 ± 0.080) than that of the SD experimental (1.743 ± 0.158) and SNT groups (2.157 ± 0.255). Differences in the weight ratio between the two groups were significant (SNT/NUDE group and SNT group, *p* = 0.0008; SNT/NUDE group and SNT/RAPA group, *p* = 0.0007). In contrast, the difference in the weight ratio between the SNT/RAPA group and the SNT group was not significant (*p* = 0.0755).

### General Observation and Histological Findings

**FIGURE 4:**
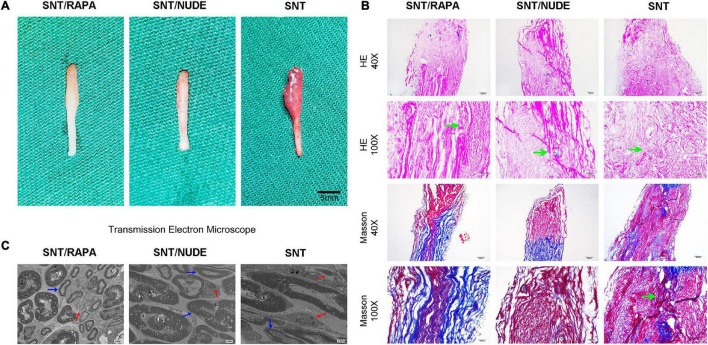
The results of gross observation and histological analysis of the PNS. **(A)** Images of neuromas in all groups. Typical globular neuromas were observed in both the SNT group and the SNT/RAPA group 30 days after the neurectomy, but no such findings were observed in the SNT/NUDE group. **(B)** H&E and Masson’s staining of neuroma 30 days after surgery. H&E staining of traumatic neuroma in the SNT/NUDE group under the light microscope, showing that the number of fibroblasts was extremely small, while the SNT/RAPA group and SNT group had a large number of fibroblasts (green arrows). Masson’s staining shows that in the SNT/NUDE group the collagen is slightly blue, and the nerve fibers are arranged relatively orderly. In the SNT group, the dense blue-stained collagen has randomly arranged nerve bundles, a large number of axons in neuromas, and more spindle fibroblasts and collagen fibers (as shown by the green arrow). Compared to the SNT group, there are fewer spindle fibroblasts and collagen fibers in the SNT/RAPA group, showing dark blue stained collagen, with regularly arranged nerve bundles. (40×, scale bar: 200um; 100×, scale bar 100 um) **(C)** Transmission electron microscopy was generally observed 30 days post-surgery. SNT/NUDE group: There are a large number of thick myelinated fibers with few fibroblasts; the red arrows indicate scattered unmyelinated fibers, and the blue arrows indicate myelinated fibers; only a few collagen fibers can be seen at random. SNT group: There are a large number of unmyelinated fibers and abundant fibroblasts; abundant horizontal and diagonal collagen fibers are randomly distributed; the marks show dense horizontal collagen fibers (red arrow) and myelin sheath (blue arrow). SNT/RAPA group: collagen fibers are distributed at the bottom; blue arrows indicate Schwann cells of internal mitochondria and endoplasmic reticulum, and red arrows indicate unmyelinated nerve fibers. (Magnification 3000×).

Thirty days after the neurectomy, typical globular neuroma was formed at the end of the trigeminal ganglion in 6 animals in the SNT group (the other 2 cases had a mild spindle-shaped stump). The SNT/RAPA group showed mild glomerular neuroma, but no glomerular neuroma was found in the SNT/NUDE group (Figure 4A). Histologically, the proximal nerve stump stained with H&E and Masson’s trichrome was depicted on the longitudinal nerve section. H&E staining revealed the following: disordered arrangement of nerve fibers in the SNT group, neuroma cells grew without tissue, regeneration of axons, muscle infiltration, formation of small sheet-like and cord-like structures by sheath cells. However, the SNT/RAPA group and the SNT/NUDE group were characterized by less proliferation of nerve fibers with a neat and regular arrangement (Figure 4B). Masson’s tricolor staining revealed that the SNT group exhibited a high proliferation of collagen (blue staining) mixed with disorderly arranged nerve bundles (Figure 4B). On the other hand, the SNT/NUDE group exhibited light blue collagen and neatly arranged nerve fibers (Figure 4B). The collagen density of SNT/RAPA group was lower than that of SNT group, and the distribution of collagen fibers was more orderly than that of SNT group, which was close to that of SNT/NUDE group (Figure 4B).

### Transmission Electron Microscope Ultrastructure Examination

The basic structure of each group comprised nerve cell membrane, Schwann cell-axon complexes, which are composed of a group of myelinated and unmyelinated axons, surrounded by collagen fibers and fibroblasts. Transmission electron microscopy demonstrated a large number of axons in the rat neuroma in the SNT group. Additionally, there were abundant horizontal and oblique collagen fibers in neuroma in SNT group, the number of fibroblasts increased and myelin sheath degenerated and proliferated. A small amount of collagen fibers and organelles, including mitochondria and endoplasmic reticulum, were seen in the nerve tissue in the SNT group. On the other hand, in the SNT/NUDE group, the thickness of the myelin sheath at the site of sciatic nerve dissection was thickened, with fewer Schwann cells, and sparse collagen fibers and fibroblasts (Figure 4C).

### The Expression of α-SMA, NF-200, and p75 in Proximal Nerve Stump

**FIGURE 5:**
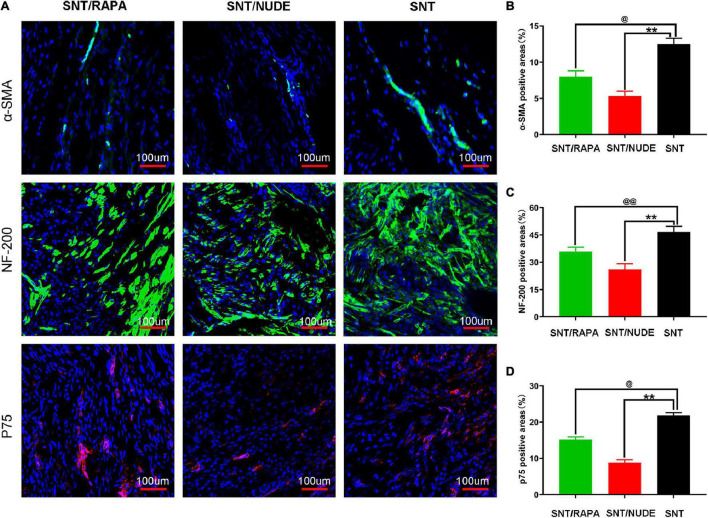
Immunofluorescence staining results of α-SMA, NF-200, and p75 in the PNS. **(A)** Representative immunofluorescence micrographs of α-SMA (green), NF-200 (green), and p75 (red) fused with DAPI (blue nuclear stain) in each group. The regenerating axons (labeled by NF-200) in the SNT group are dense and chaotic. The larger regenerating axons in the SNT/RAPA group are arranged more regularly than in the SNT group. Only a few axons (labeled by NF-200) are regularly arranged linearly in the SNT/NUDE group. (100×, scale bar: 100 um) **(B–D)** Quantification of **α**-SMA, NF-200 and p75 positive areas in each group (^@^*p* < 0.05, ^@@^*p* < 0.01, **p < 0.01). Data are expressed as mean ± SEM, *n* = 6 rats per group. **α**-SMA (SNT group: 12.500 ± 0.462; SNT/RAPA group: 8.007 ± 0.526; SNT/NUDE group: 5.320 ± 0.387); NF-200 (SNT group: 46.700 ± 1.735; SNT/RAPA group: 35.767 ± 1.433; SNT/NUDE group: 26.000 ± 1.825); p75 (SNT group: 21.800 ± 0.473; SNT/RAPA group: 15.200 ± 0.416; SNT/NUDE group: 8.833 ± 0.441).

The potential role of regulatory T cells in preventing the formation of traumatic neuroma and neuropathic pain was explored using the rat model of sciatic nerve dissection and give immunosuppressive treatment. Immunofluorescence single staining results showed that, on the 30^th^ day after the surgery, the immunoreactivity of a-SMA (α smooth muscle actin) and p75 (nerve growth factor receptor) in the SNT/RAPA group were significantly lower a (*P* < 0.05) as compared to that of the SNT group. The SNT/NUDE group expressed the lowest a-SMA and p75 levels (*P* < 0.01). In addition, the immunoreactivity of NF-200 (neurofilament, axon marker) in the SNT/NUDE group was significantly lower than that in the SNT group (*P* < 0.01), and NF-200 expression in the SNT/RAPA group was decreased (*P* < 0.01) (Figures 5A–D). These findings are consistent with the results of the previous morphological analysis of nerves.

### Differentiation of M1/M2 Macrophages in Dorsal Root Ganglion

**FIGURE 6:**
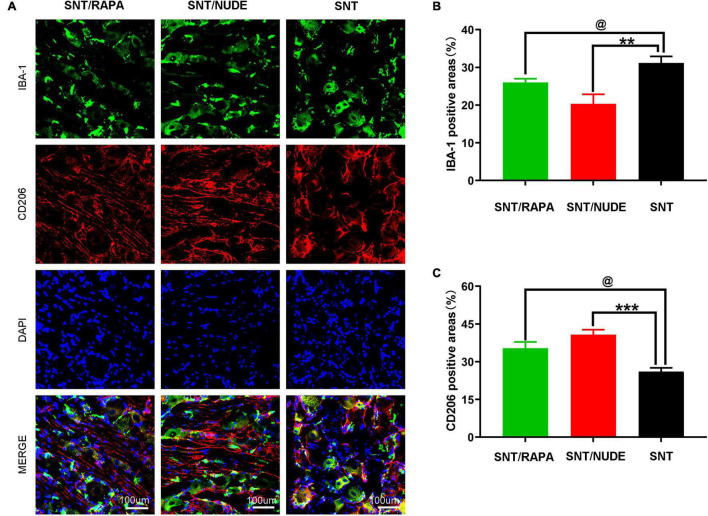
Immunofluorescence staining results of M1 and M2 macrophages in DRG. **(A)** Representative photomicrographs of IBA-1 (green) and CD206 (red) immunofluorescence in each group. DAPI: Nuclear staining (blue). (100×, scale bar: 100um) **(B,C)** Quantification of IBA-1 and CD206 positive areas in each group (^@^*p* < 0.05 **p < 0.01, ***p < 0.001). Data are expressed as mean ± SEM, *n* = 6 rats per group. IBA-1: (SNT group: 31.167 ± 1.014; SNT/RAPA group: 26.001 ± 0.577; SNT/NUDE group: 20.333 ± 1.453); CD206: (SNT group: 26.033 ± 0.895; SNT/RAPA group: 35.333 ± 1.453; SNT/NUDE group: 40.700 ± 1.179).

Macrophages in the parallel DRG play a critical role in the initiation and maintenance of the mechanical hypersensitivity of the neuropathic pain phenotype after peripheral nerve injury. Notably, there is a potential correlation between the polarization direction of macrophages in DRG and neuroinflammation and neuropathic pain. In this view, through the immunofluorescence double staining method, we detected the presence of M1 and M2 macrophages in DRG at 30 days after neurotomy. Results demonstrated that, compared to the SNT group, the immunoreactivity of M1 macrophages in the DRG of the SNT/NUDE group was significantly reduced (*P* < 0.01), whereas the immunoreactivity of M2 macrophages was significantly increased (*P* < 0.001), with significantly lower M1/M2 ratio. In the SNT/RAPA group managed by immunosuppressive therapy, the immunoreactive area of M1 macrophages was decreased (*P* < 0.05), the immunoreactive area of M2 macrophages was increased (*P* < 0.05), and the proportion of M2/M1 was lower than that of SNT/NUDE group (Figures 6A–C). The DRG under lower multiples (20×) can be seen in our Supplementary Figure 1.

### Thermal Pain Measurement and Pain-Related Protein Expression

**FIGURE 7:**
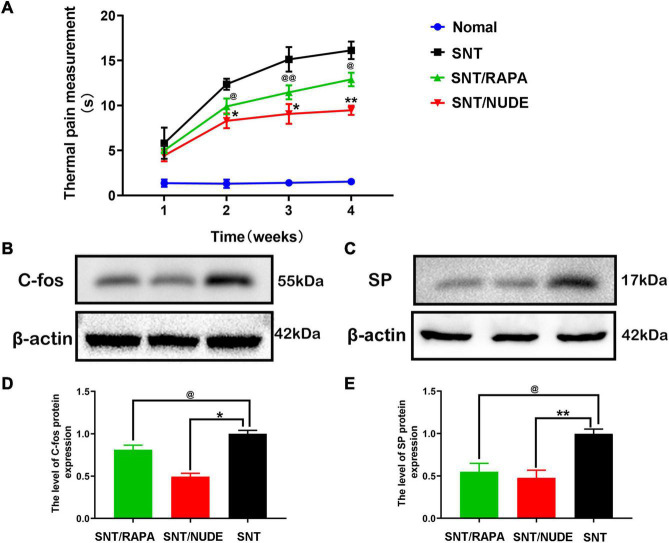
Results of thermal pain measurement and expression of pain-related proteins c-fos and SP. **(A)** Weekly thermal pain response time measurement results. Except for the first week, the thermal pain response time between the SNT group and the other two groups was statistically different at all time points (*p < 0.05,^ **^p < 0.01, ^@^*p* < 0.05 ^@^^@^*p* < 0.01). **(B,D)** The expression result of c-fos in the spinal cord. As a pain marker, the expression of c-fos in the L4 lumbar spinal cord DRG was lower in the SNT/RAPA group than in the SNT group (^@^*p* < 0.05), and the expression in the SNT/NUDE group was significantly lower than that in the SNT group (*p < 0.05). **(C,E)** The result of the expression of substance p in the nerve stump. The content of substance p in the SNT group was significantly higher than the other two experimental groups (^@^*p* < 0.05, **p < 0.01).

At 1, 2, 3, and 4 weeks post-neurectomy, the hot and cold pan pain meter [BIO-CHP] was utilized to measure and record the thermal pain response time of rats in each group. The same technician recorded the response time of each group of experimental rats raising their forelimbs and compiled the data into an intuitive line chart (Figure 7A). With the extension of time after neurotomy, the results of thermal pain measurement showed that compared with normal rats, the time of lifting front paws of rats in each experimental group was gradually prolonged, and the difference was statistically significant (*p* < 0.05). Except for the first week, compared to the SNT/NUDE group (9.467 ± 0.513, the 4^th^ week), the thermal pain response time of the SNT group (16.133 ± 0.971, the 4^th^ week) was significantly longer (*p* < 0.05), while that of the SNT/RAPA group (12.900 ± 0.755, 4^th^ week) was also significantly longer than that of SNT/NUDE group (*p* < 0.05). On the 30^th^ day post-surgery, western blot detection showed that compared to the SNT group, c-fos expression in the fourth lumbar spinal dorsal horn of the SNT/RAPA group was decreased significantly (*p* < 0.05), similarly, c-fos expression in the SNT/NUDE group was also decreased significantly (*p* < 0.05). At the same time, the level of substance P in the nerve stump of the SNT/NUDE group was significantly lower than that in the SNT group (*p* < 0.01). Similarly, the expression of substance P in the SNT/RAPA group was lower as compared to that in the SNT group (*p* < 0.05) (Figures 7B–E).

### Biological Safety Toxicity Test in Rats

**FIGURE 8:**
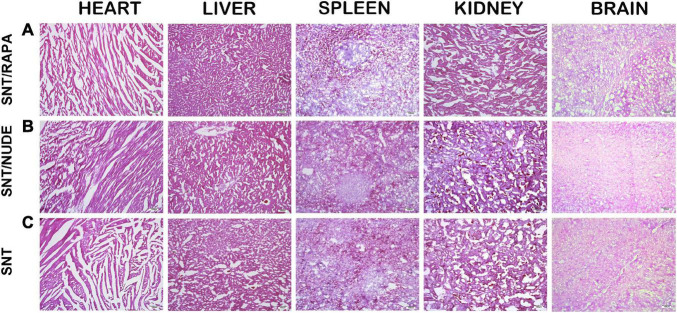
Biosafety toxicity test results in rats (to evaluate whether the drug treatment has a toxic pathological effect on the biosafety of rats). Thirty days after surgery, the main organs (heart, liver, spleen, kidney, brain) of rats in all groups were sectioned and H&E stained. The results showed no obvious structural difference between the groups (100×, scale bar: 100 um).

No noticeable evidence of biological toxicity or animal deaths were reported in the entire study. The SNT group, the SNT/RAPA group, and the SNT/NUDE group were not significantly different in terms of body weight, food consumption, and food utilization. The main organs of each group of experimental rats were analyzed through H&E staining after different treatments. The H&E staining results demonstrated that compared to the SNT group, the major organs and tissues (heart, liver, spleen, kidney, brain) of rats in the SNT/NUDE group and the SNT/RAPA group treated with immunosuppressants exhibited no obvious structural lesions and toxicity change (Figure 8).

## Discussion

Transection of the peripheral nerve causes an inevitable Wallerian degeneration at the distal end of the lesion. Such a rapid degeneration process begins with the degradation of axoplasm and axons, resulting in axon and myelin fragments, which are then cleared by Schwann cells and invading macrophages. After Wallerian degeneration, the regenerated axons at the proximal end grow into the surrounding normal tissues causing abnormal growth, tangling of the regenerated axons and forming dense nerve masses called a neuroma (Stoll et al., 2002; Beirowski et al., 2005; Galeano et al., 2009). In most cases, a painful neuroma is a severe sequelae caused by peripheral nerve injury but the precise pathophysiological mechanism through which it is formed is elusive (Hirose et al., 2010; Watson et al., 2010). Many authors have reported the successful prevention and treatment of painful neuromas after the traumatic neuromas were isolated from the scar tissues and transferred to the surrounding muscles, veins, or bones. So far, no consensus has been reached regarding the best treatment approach for a painful neuroma (Burchiel et al., 1993; Balcin et al., 2009; Yan et al., 2012). As such, neuropathic pain remains a major problem in the field of pain and is a huge challenge for clinicians (Rowbotham, 2005).

Macrophages (Mφ) are important members of innate immunity. Previously the major research focus has been on the origin and function of macrophages and their promotion or protection effects on diseases (Funes et al., 2018). Within the microenvironment, multiple cytokines particularly induce the polarization of macrophages into M1/M2 type macrophages which express corresponding specific genes and perform different functions (Figure 1) M1 type macrophages (classically activated macrophages, AMs) mainly exert pro-inflammatory, bactericidal, and antigen presentation roles. M2 type macrophages (alternatively activated macrophages, AAMs) secrete anti-inflammatory cytokines, etc., critical in the anti-parasitic immune response and play vital roles in allergy, damage repair, and tissue remodeling (Figure 1). The peripheral ending of a DRG neuron with the cell body situated in a DRG is activated and the action potential (nerve signal) passes from the peripheral to the central process via the T-junction (Esposito et al., 2019). Several studies have demonstrated that macrophages in DRG contribute to the initiation and persistence of neuropathic pain, and are also indispensable in the initiation and maintenance of the mechanical hypersensitivity response to the neuropathic pain phenotype (Kazuhide and Makoto, 2009; Chen et al., 2018; Yu et al., 2020). Dorsal root ganglion as a potential new peripheral target of neuromodulation, remains a feasible choice for the treatment of chronic intractable neuropathic pain.

Previous investigation in our laboratory revealed the aggregation of several types of macrophages in the DRG of the spinal cord of ordinary SD rats at different stages of injury, implying a higher ratio of M1/M2. However, it remains to be determined whether this phenomenon is related to neuropathic pain caused by the traumatic neuroma. Mounting evidence shows that peripheral monocytes and macrophages play an active role in pain via different mechanisms (Xu et al., 2010; Willemen et al., 2014; Ghasemlou et al., 2015). Several lines of evidence have demonstrated the role of T lymphocytes in neuropathic pain. For instance, after nerve injury, T cells infiltration into the DRG induces the release of the pro-pain mediator leukocyte elastase (LE), causing mechanical hyperalgesia (Liu et al., 2014; Vicuña et al., 2015). Similarly, in the present study, a sciatic nerve dissection model of the nude rats (a mutant rat with congenital thymic T cell defects) revealed a low M1/M2 ratio in spinal cord DRG. A comprehensive pain assessment of the SNT/NUDE group 30 days after the neurotomy demonstrated that the rats exhibited significantly lower pain and did not have a traumatic neuroma. Therefore, it was suggested that the expression of T cell subsets-regulatory T cells (Tregs) may directly influence the polarization direction of macrophages in rat DRG, and play a specific regulatory role in neuropathic pain. In this view, SD rats were managed by immunosuppressive therapy (intraperitoneal injection of rapamycin) after nerve dissociation and monitored. Results revealed that compared to the SNT group, the immune response activity of M1 macrophages in the animal DRG of the SNT/NUDE group was significantly decreased, whereas the immune response activity of M2 macrophages was significantly increased, and the ratio of M1/M2 was significantly lower. These findings strongly demonstrate a role for immunosuppressive therapy to effectively reduce the expression of regulatory T cells in rats subjected to sciatic nerve dissection. This management approach can promote the polarization of macrophages in the DRG of the rat spinal cord to M2 and decreases the production of M1 macrophages.

In clinical practice, the pain state of patients can be easily expressed by words. However, animal experiments lack an effective method for assessing whether experimental traumatic neuropathic pain is relieved (Sakai et al., 2005; Butler et al., 2007). Animals that produce autotomy behavior has been considered as an animal model of spontaneous sensory disturbances related to neuropathic pain, such as anesthesia pain and phantom limb pain (Wall et al., 1979; Coderre et al., 1986). In the present study, compared to the SNT group, the autotomy behavior of the SNT/RAPA group and the SNT/NUDE group was significantly inhibited after the 5^th^ experimental day. However, no significant difference in the autotomy scores was reported between the two experimental groups. Evidence on whether autotomy behavior is directly related to pain is lacking (Sakai et al., 2005). Quantitative assessment of tactile abnormalities (Chaplan et al., 1994) or Hargreaves plantar thermal hyperalgesia test (Hirose et al., 2010) is suggested as the best method for assessing neuropathic pain of traumatic neuroma. Herein, we accurately examined the pain state of neuroma and studied the autotomy behavior of rats using the hot and cold pan pain meter to measure and record the thermal pain response time of rats in each group. Results were statistically significant, showing that the time of raising the front paws of rats in each experimental group gradually increased with time after nerve transection compared to normal rats. Except for the first week, the thermal pain response time of the SNT group was significantly longer compared to that in the SNT/NUDE group, while the thermal pain response time of the SNT/RAPA group was significantly lower than that of the SNT group. The walking footprints of each group of rats recorded in real-time by CatWalk XT on the 30^th^ day after surgery corroborated the findings obtained from the side. The gait imprints of the rats in the two experimental groups, particularly the SNT/NUDE group were closer to that of normal rats. Rats in the SNT group exhibited decreased gait speed and gait frequency influenced by pain. Several literature reports have investigated the expression levels of pain-related protein c-fos and substance P (Siddall et al., 1999; Lindqvist et al., 2000; Zochodne et al., 2001; Sakai et al., 2005). Findings from these studies shows that the expression of c-fos proto-oncogene in the nucleus of spinal cord postsynaptic neurons is a crucial indicator of neural activity and pain caused by severance, ligation, chemical stimulation, and allodynia (Kajander et al., 1996; Siddall et al., 1999). Substance P and calcitonin gene-related peptide-positive nerve fibers are also abundant in mechanically sensitive human neuromas and are suggested to contribute to the mechanisms of local pain (Lindqvist et al., 2000). The present investigation revealed significantly lower expression levels of c-fos and SP in the SNT/RAPA group than that in the SNT group. Intriguingly, the expression levels of c-fos and SP in the SNT/NUDE group were lower than those in the SNT/RAPA group, providing more evidence on the regulatory role of regulatory T cells in suppressing neurotomy-induced pain. The results suggest that the regulatory effect of regulatory T cells on the M2-type polarization of macrophages holds great promise to prevent neuropathic pain associated with a traumatic neuroma.

Evidence shows that α-smooth muscle actin (α-SMA) is a useful phenotypic marker of myofibroblasts (MFB) and may enhance their contractile activity and trigger neuroma-related pain (Yan et al., 2012; Weng et al., 2016). The low-affinity receptors of nerve growth factor p75 and NF-200 are thought to be associated with cell proliferation, myelination, and synaptic plasticity after peripheral nerve injury. Previous evidence has confirmed high expression of NF-200 and p75 in painful neuroma, which is maintained for a long time (Yan et al., 2014; Zhou et al., 2020). Therefore, the present work, utilizing immunofluorescence, assessed the expression of α-SMA, NF-200, and p75 in the PNS. Results showed that, compared to the SNT group, the expression of a-SMA and p75 in the SNT/RAPA group was significantly decreased, and that in the SNT/NUDE group was the lowest. NF-200 expression in the SNT/NUDE group was significantly lower than that in the SNT group, and the expression in the SNT/RAPA group was also reduced significantly compared to that in the SNT group. The findings demonstrate that the regulatory effect of regulatory T cells on the M2-type polarization of macrophages can alleviate neuropathic pain and play a role in the regulatory process of neuroma formation via a defined mechanism.

Neurotrophic factors spread from all directions and attract regenerated nerve fibers, resulting in irregularly distributed nerve fibers closely mixed with proliferated connective tissue to form spherical neuromas (Stoll and Muller, 1999; Watson et al., 2010; Rajput et al., 2012). H&E staining and Masson’s staining results from the present work also proved the existence of this phenomenon in the proximal stump of the SNT group. Moreover, the regenerated nerve fibers in the SNT/RAPA group and the SNT/NUDE group were more regular than those in the SNT group. Studies have demonstrated that the ratio of myelinated fibers to unmyelinated fibers is a crucial pathological characteristic of a traumatic neuroma, which is determined by the function of Schwann cells (Wall and Gutnick, 1974; Carlton et al., 1991). Generally, painful neuroma regenerating nerve fibers manifests as countless small nerve bundles, in which their unmyelinated fibers are much more than myelinated fibers (Cravioto and Battista, 1981). In the present study, transmission electron microscopy revealed fewer unmyelinated fibers in the SNT/RAPA group and SNT/NUDE group than in the SNT group, with more thick myelinated fibers. Pain is mainly mediated by unmyelinated nerve fibers (COLLINS et al., 1960; Zimmermann, 2001), therefore, the findings provide evidence that the regulatory effect of regulatory T cells on the M2 polarization of macrophages also plays a part in preventing the formation of painful neuromas.

In summary, the differentiation of the M1/M2 type of macrophages on the DRG of the rat spinal cord plays a significant role in blocking the afferents of various types of peripheral neuropathic pain after neurotomy. By inhibiting the expression of regulatory T cells in rats, immunosuppressive therapy promotes M2-type polarization of macrophages and reduces the production of M1-type macrophages. As a result, the afferents of peripheral neuropathic pain are intercepted and subsequently block the formation of painful neuromas. The findings provide concrete evidence to guide the development of new strategies for painful neuroma management. Our subsequent research will focus on elucidating the key upstream control molecules or the key targets of the regulatory network that promote M2 polarization and inhibit M1 conversion in response to this phenomenon. Timely regulation of the target microglia/macrophage phenotype can offer the best treatment approach for painful neuroma, and guide future clinical work.

## Conclusion

M1/M2 type differentiation of macrophages on DRG plays a pivotal role in the formation of traumatic neuroma after neurotomy. In particular, regulatory T cells may reduce the ratio of M1/M2 types of macrophages by mediating the polarization direction of macrophages on the DRG. In this view, the input of peripheral nerve pain is cut off, which benefits nerve damage repair. The findings may provide concrete evidence to guide the development of new strategies for managing painful neuroma.

## Data Availability Statement

The original contributions presented in the study are included in the article/Supplementary Material, further inquiries can be directed to the corresponding authors.

## Ethics Statement

This study involving animals was reviewed and approved by The Animal Care Committee of Wenzhou Medical University, China (Approval: wydw2019-0954). All experiments involving animals were conducted according to the ethical policies.

## Author Contributions

All authors made substantial contributions to conception and design, acquisition of data, or analysis and interpretation of data; took part in drafting the article or revising it critically for important intellectual content; agreed to submit to the current journal; gave final approval of the version to be published; and agreed to be accountable for all aspects of the work.

## Conflict of Interest

The authors declare that the research was conducted in the absence of any commercial or financial relationships that could be construed as a potential conflict of interest.

## Publisher’s Note

All claims expressed in this article are solely those of the authors and do not necessarily represent those of their affiliated organizations, or those of the publisher, the editors and the reviewers. Any product that may be evaluated in this article, or claim that may be made by its manufacturer, is not guaranteed or endorsed by the publisher.

## Supplementary Material

Supplementary Figure 1 The DRG under lower multiples (20×).

Supplementary Figure 2 T cells and M2 macrophages in the sciatic nerve.

## Abbreviations

DRG: dorsal root ganglion
Tregs: regulatory T cells
SD: Sprague Dawley
α-SMA: alpha-smooth muscle actin
NF-200: neurofilament 200
M φ: Macrophages
WR: weight ratio
NW: weight of neuroma
NNW: weight of normal nerve segment removed
H&E: Hematoxylin and eosin
IOD: the integrated option density
SDS-PAGE: sodium dodecyl sulfate-polyacrylamide gel electrophoresis
PVDF: polyvinylidene fluoride
SP: substance P
β-actin: beta-actin
PNS: proximal nerve stump
LE: leukocyte elastase
MFB: myofibroblasts.
