# Supplementary figures and images for: Exploring the Correlation Between the Regulation of Macrophages by Regulatory T Cells and Peripheral Neuropathic Pain
Source: Front Neurosci. 2022 Feb 14;16:813751. doi: 10.3389/fnins.2022.813751 (PMC8882923; doi:10.3389/fnins.2022.813751)

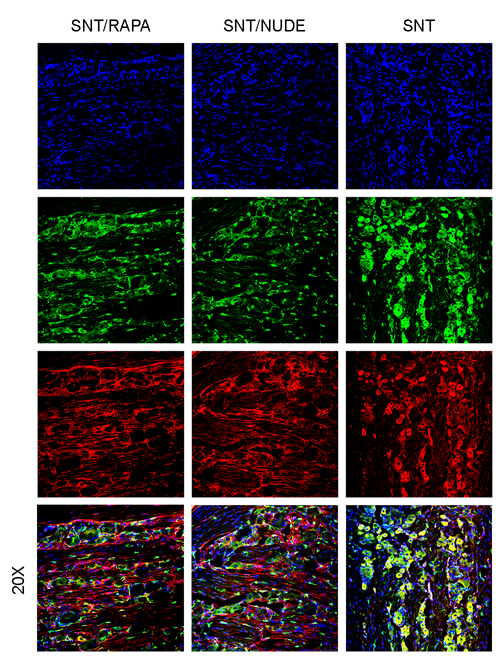

Supplement: Supplementary Figure 1 — The DRG under lower multiples (20×). [file Image_1.tif]

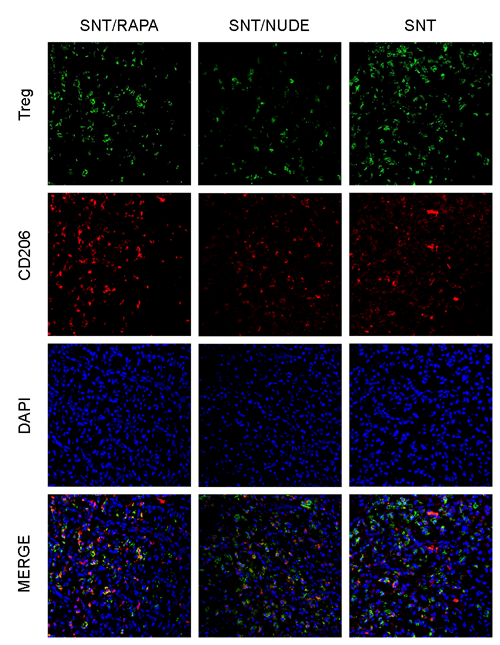

Supplement: Supplementary Figure 2 — T cells and M2 macrophages in the sciatic nerve. [file Image_2.tif]
